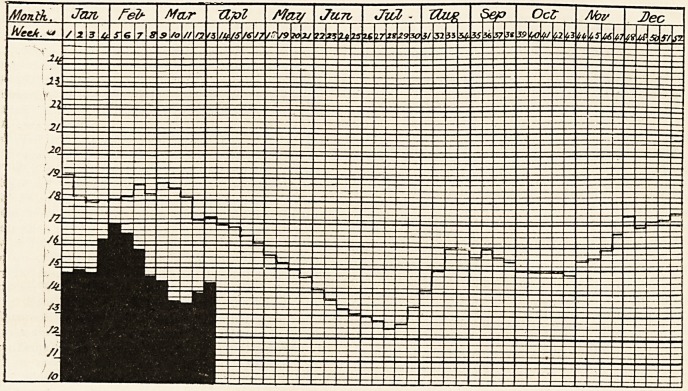# Diagram of the Weekly Death-Rate in 1910

**Published:** 1910-05-07

**Authors:** 


### Public Health and Hygiene.

### DIAGRAM OF THE WEEKLY DEATH-RATE IN 1910.

Showing the weekly death-rate for 1910 according to the Registrar-General and the mean weekly death-rate for the last eight years of the 76 * great towns of England and Wales.

White columns show mean weekly death-rate for last eight years. Black columns show weekly death-rate for current year Where the death-rate for 1909 is in excess of the eight-yearly mean, the excess is shown in black above the white column which represents the mean. Where the death-rate for 1910 is below the eight-yearly mean, the black column is shown in its entire length; the white column, which represents the mean, showing above the black.
Where the death-rate for 1909 coincides with the eight-yearly mean, it is shown thus, xx.
* Since March 31, 1910, 77 great towns.

## Figures and Tables

**Figure f1:**